# Gender differences in suicidal ideation and health-risk behaviors among high school students in Beijing, China

**DOI:** 10.7189/jogh.09.010604

**Published:** 2019-06

**Authors:** Yi-Yang Zhang, Yuan-Ting Lei, Yi Song, Ruo-Ran Lu, Jia-Li Duan, Judith J Prochaska

**Affiliations:** 1 Peking University Third Hospital, Peking University, Beijing, China; 2 Institute of Child and Adolescent Health, School of Public Health, Peking University, Beijing, China; 3 Stanford Prevention Research Center, Department of Medicine, Stanford University, Stanford, California, USA; 4 Beijing Center for Disease Prevention and Control, Beijing, China

## Abstract

**Background:**

Suicide is still the leading cause of death in the 15 to 34-year age group, especially for girls aging 15 to 19-year old. In China particularly, the suicide rate of female is 60% higher than male. The gender difference on suicidal ideation and its patterns with academic, family, social and health-risk factors is unknown among adolescents in Beijing, China.

**Methods:**

A total of 33 635 students in grades 7-12 in Beijing participated in the 2014 Chinese Youth Risk Behavior Surveillance. Data were stratified by gender and associations with suicidal ideation were analyzed using χ^2^ test and multivariate regression analyses. The interaction effects on suicidal ideation between gender and the related behaviors were also analyzed.

**Results:**

The prevalence of suicidal ideation was significantly higher for girls (13.3%) than boys (10.7%). The multivariate regression analyses indicated that high academic pressure, running away from home, feeling lonely or sad/hopeless, being bullied, fighting, and binge drinking were significantly associated with suicidal ideation in boys and girls. Factors more strongly associated with suicidal ideation in girls than boys were being in junior vs senior high school (girl vs boys: 1.24 vs NA), high academic pressure (2.42 vs 1.55), ever smoking (1.52 vs NA), binge drinking (1.30 vs 1.17), fighting once (1.63 vs 1.06) and being sad/hopeless (2.39 vs 2.04) and their interaction with gender were all statistically significant (*P* < 0.05). A lower likelihood of suicidal ideation was found among boys, but not girls, who had PE class two or more days per week.

**Conclusions:**

Girls showed more vulnerability to suicidal ideation than boys particularly among girls in junior school, reporting high academic pressure, smoking, binge drinking and fighting. The combinations of risk factors and differential patterns for boys and girls point to high-risk groups and potential targets for gender-specific suicide prevention.

Globally, over 800 000 die of suicide every year and there is an estimation that for each individual who dies of suicide there may have been more than 20 others attempting suicide [1]. Although suicide prevalence varies among different countries and regions, the World Health Organization [2] reported that suicide was the second leading cause of death in the 15 to 29-year age group in 2014. Moreover, among girls ages 15 to 19 years old, suicide has become the leading cause of death globally.

Although the prevalence of suicide in China decreased from 15.68 to 8.14 per 100 000 between 2002 and 2011 [3], suicide remains the leading cause of death in the 15 to 34-year age group [4]. China’s One-Child Policy resulted in most students being the only child in their family. Childhood suicide brings significant trauma to the family and has been recognized as a serious public health problem. By 2010, the official estimate of the annual number of deaths of only-children aged 15–30 was 76 000, and the total number of Chinese families who have lost their only-children (aged over 30 were included) was approximately 1 million [5]. It is expected that this figure will reach 10 million by 2035 [6].

Many studies have reported gender differences in suicide behaviors [7-9], with an overrepresentation of females reporting suicidal ideation and exhibiting nonfatal suicidal behavior and a preponderance of males completing suicide, which is known as the “gender paradox of suicidal behavior” [10]. In developed countries, the risk of suicide is two (Western Europe) to 4-fold (USA) greater for males than females [11]. Worldwide, more than twice as many males than females aged 15 to 19 years commit suicide (4.1 per 100 000 for females and 10.5 per 100 000 for males) [12].

Different cultural backgrounds have different effects on suicidal behaviors. A higher completed suicide prevalence among teenage girls than boys is evident in countries such as China and India (particularly southern India) compared to the Western world [13-15]. This may in part be attributed to intergenerational and gender conflicts being more distilled and pronounced in traditional agricultural societies that are emerging into egalitarian industrial societies [16]. In China particularly, the pattern of youth suicides differs greatly to that in the West: female rate is 60% higher than male rate and rural rate is 3-fold urban rate [17].

Suicide behaviors have been shown to be associated with a number of risk behaviors that in turn may also have immediate and long-term adverse health consequences. For example, pre-teen alcohol use initiation is associated with suicide attempts in young people and with girls at a higher risk compared with boys [18]. An analysis of data on US high school students from 1991-2011 identified five dimensions associated with suicidal thoughts and attempts; they were: violence (community or school related), substance use, sexual health, weight-related, and physical/sedentary activities [19]. The findings were recommended to promote school-based suicide prevention programs.

Most studies of youth suicidal ideation and behavior in China have been regional with small and non-representative samples. In a large representative sample, the current study sought to: (1) describe the prevalence of suicidal ideation among boys and girls in Beijing, China; (2) examine the association between suicidal ideation with family, academic and social factors and health-risk behaviors; and (3) examine gender differences in the patterns of association with suicidal ideation. The variables of interest were informed by the literature and included family structure, running away, bullying victimization and violence, mental health problems, substance use behaviors (tobacco and alcohol), and physical activities.

## METHODS

### Design and participants

A cross-sectional survey of 33 694 middle school students from Beijing, China was conducted. Peking University’s Medical Research Ethics Committee (IRB00001052-17010) approved the study procedures. Informed consent was obtained from parents and assent from the students. All of the respondents were informed that the survey would be conducted anonymously, and that their privacy would thus be respected.

A three-stage, stratified sampling method was used to obtain a representative sample of students in grades 7-12 in Beijing. First, three kinds of districts or counties were sampled according to their socioeconomic level (upper, moderate, lower). Second, the junior and senior high schools were categorized as vocational, ordinary, or key (ie, those with good records of past educational accomplishment had priority in the assignment of teachers, equipment, and funds and the privilege of recruiting the best students). Based on probability proportional to school enrolment size, we selected 31 vocational senior high schools (grade 10-12), 36 ordinary junior high schools, 35 ordinary senior high schools, 27 key junior high schools, and 36 key senior high schools. Finally, we used a simple random sampling method to select n classes from each grade at each school (n depended on the average size of classes and was no less than 200 students per school). The survey was launched between April and May 2014. Participants completed the self-administered anonymous questionnaire in their classroom in the absence of school teachers

### Measures

The questionnaire was derived from the Chinese Youth Risk Behavior Surveillance (CYRBS) survey in 2014, which assesses six categories of priority health-risk behaviors contributing to the leading causes of death, disability, and social problems. The CYRBS survey [20] was adapted from the 2003 Youth Risk Behavior Surveillance survey in the United States, for which a high degree of reliability and validity has been documented previously [21-23]. The CYRBS questionnaire was reviewed by education and health experts and pilot-tested in Beijing and Jinan [24]. Suicidal ideation was assessed with the question “During the past 12 months, did you ever seriously consider attempting suicide?” Response categories were “Yes” or “No.” **Table 1** summarizes the academic, family, social and health-risk behavior CYRBS items of interest for the current analyses. In addition, school grade was categorized as junior or senior high school; school type as vocational, ordinary or key; and gender as boys or girls.

**Table 1:** Characteristics of independent variables

Construct	Description of questions	Responses
Academic pressure	In the past 12 months, how often did you feel unhappy because of stress/pressure from school?	“none” = ”low”, “rarely or sometimes” = “average”, “often or always” = “high”
School achievement	How do you think your performance compares with your classmates?	“above average”, “average”, “below average” “not sure
Family structure	Who do you live with at home?	“both parents” vs other
Smoking	Have you ever tried cigarette smoking, even one or two puffs	“Yes” or “No”
Binge drinking last 30 days	During the past 30 days, on how many days did you have five or more drinks of alcohol in a row that is, within a couple of hours?	“0” or “1-30”
Physical fighting past 12 months	During the past 12 months, how many times were you in a physical fight	“0”, “1” or “2 or more times”
PE classes	In an average week when you are in school, on how many days do you go to physical education (PE) classes	“0 or 1 day” and “More days”
Exercising	During the past 7 days, on how many days were you physically active for a total of at least 60 min per day?	“0-2 days” and “More days”
Considered running away from home in last 12 months	During the past 12 months, did you ever seriously consider running away from home that is, leaving your home for 24 h or more without your parents’ permission?	“Yes” or “No”
Ran away from home in last 12 months	During the past 12 months, did you ever attempt to run away from home (for 24 h or more without your parents’ permission)?	“Yes” or “No”
Victim of bullying	During the last 12 months have you ever been (a) ‘hit, kicked, pushed, shoved around, or locked indoors?’ (b) ‘Made fun of or insulted?’ (c) ‘Excluded intentionally or prevented from participating?’ (d) ‘Made fun of with gender jokes, comments or gestures?’; ‘blackmailed for money?’ or (f) ‘bullied in some other way?’	Victims who reported at least one victimization experience in the last 12 months with a frequency of “often”
Feeling lonely	During the past 12 months, did you ever feel lonely	“Never”, “rarely or sometimes” and “often or always”
Feeling sad/hopeless	During the past 12 months, did you ever feel so sad or hopeless almost every day for 2 weeks or more in a row that you stopped doing some usual activities	“Yes” or “No”

### Statistical analysis

Of all 33 694 students, 33 635 (99.8% of the sample) were included in the analyses. 59 of all 33 694 records were excluded because the information of suicide ideation was missing. Of them, 13 were junior school students and 46 were from senior high school. The gender of the missing data was evenly distributed. **Table 2** summarizes the sample characteristics of suicidal ideation prevalence overall and by gender. We examined the prevalence of suicidal ideation for boys and girls and the association with violence-related behaviors, substance use behaviors, physical activities, mental health problems and running away from home by χ^2^ test. Those variables significantly related with suicide ideation in univariate analysis were included in multivariate logistic regression model and analyzed as co variables by the method of Backward wald. Multivariate logistic regression analyses were stratified by gender, and school type was adjusted in all models. For boys, multivariate logistic regression analyses were limited to the 91.6% (15 428/16 850) boys for which complete information was available, and 8.4% (1422/16 850) records were excluded because one or more independent variables were missing, and for girls, 93.7% (15 733/16 785) records were included and 6.3% (1052/16 785) were excluded due to missing information of one or more independent variables. We examined adjusted OR and 95% confidence intervals (CI) to assess the significance of the associations. The percent relative standard error (PRSE) test was performed for testing the reliability of regression coefficients. PRSE<25% could be considered as a reliable estimation [25]. Besides, interaction analysis was used to test the between-groups difference of gender. All analyses were conducted using SPSS 19.0 (IBM, Armonk, New York, USA). Two-sided *P* values of <0.05 were considered significant.

**Table 2:** Characteristics of suicidal ideation prevalence by genders

Variables		Total/Count (%)	Boys			Girls		
		**N**	**Count (%)**	*P*-value*	**N**	**Count (%)**	*P*-value*
Grade	Junior (grade 7-9)	14 844 (11.7)	7708	747 (9.7)	<0.001	7136	992 (13.9)	0.042
	Senior (grade 10-12)	18 549 (12.2)	8996	1041 (11.6)		9553	1225 (12.8)	
School type	Key	12 391 (12.2)	5946	659 (11.1)	0.200	6445	848 (13.2)	0.815
	Ordinary	13 197 (11.7)	6625	679 (10.2)		6572	861 (13.1)	
	Vocational	5202 (12.4)	2776	313 (11.3)		426	330 (13.6)	
Family structure	Both parents	28 121 (11.1)	13 983	1372 (9.8)	<0.001	14 138	1746 (12.3)	<0.001
	Others	5514 (16.6)	2867	431 (15.0)		2647	485 (18.3)	
Academic pressure	Low	6258 (6.6)	3969	267 (6.7)	<0.001	2289	145 6.3)	<0.001
	Average	18 568 (8.3)	8578	651 (7.6)		9990	894 (8.9)	
	High	8788 (23.6)	4293	885 (20.6)		4495	1191 (26.5)	
Academic achievement	Above average	12 389 (10.5)	5741	547 (9.5)	<0.001	6648	756 (11.4)	<0.001
	Average	10 315 (10.6)	4792	415 (8.7)		5523	674 (12.2)	
	Below average	8637 (15.3)	4954	655 (13.2)		3683	665 (18.1)	
	Not sure	1638 (14.7)	962	141 (14.7)		676	100 (14.8)	
Ever smoking	Yes	7686 (19.1)	4989	764 (15.3)	<0.001	2697	706 (26.2)	<0.001
	No	25 414 (9.9)	11 554	1007 (8.7)		13 860	1504 (10.9)	
Binge drinking last 30 d	Yes	3855 (22.7)	2612	497 (19.0)	<0.001	1243	377 (30.3)	<0.001
	No	29 693 (10.6)	14 183	1303 (9.2)		15 510	1851 (11.9)	
Fighting	Never	26 953 (9.9)	11 759	944 (8.0)	<0.001	15194	1739 (11.4)	<0.001
	Once	3064 (16.5)	2206	266 (12.1)		858	241 (28.1)	
	Twice or more	3587 (23.3)	2869	590 (20.6)		718	247 (34.4)	
PE classes	0 or 1 per week	2200 (15.3)	1135	181 (15.9)	<0.001	1065	156 (14.6)	0.173
	≥2 per week	31 309 (11.7)	15 647	1611 (10.3)		15 662	2065 (13.2)	
Exercising ≥60 min	0-2 days per week	10 568 (13.0)	4206	493 (11.7)	0.013	6362	879 (13.8)	0.116
	>2 days per week	23 055 (11.5)	12 637	1309 (10.4)		10 418	1351 (13.0)	
Considering running away from home	Yes	9686 (27.2)	4377	1140 (26.0)	<0.001	5309	1492 (28.1)	<0.001
	No	23 448 (5.7)	12 187	631 (5.2)		11 261	707 (6.3)	
Running away from home	Yes	1969 (37.6)	1187	425 (35.8)	<0.001	782	315 (40.3)	<0.001
	No	31 500 (10.3)	15584	1360 (8.7)		15 916	1887 (11.9)	
Victim of bullying	Yes	3902 (25.6)	2732	611 (22.4)	<0.001	1170	389 (33.2)	<0.001
	No	29 580 (10.1)	14 034	1177 (8.4)		15 546	1825 (11.7)	
Feeling lonely	Never	11 048 (5.0)	6253	304 (4.9)	<0.001	4795	252 (5.3)	<0.001
	Sometimes or seldom	18 100 (11.0)	8396	859 (10.2)		9704	1138 (11.7)	
	Often or always	4461 (33.1)	2188	638 (29.2)		2273	840 (37.0)	
Being sad/hopeless	Yes	4537 (30.3)	2489	654 (26.3)	<0.001	2048	725 (35.4)	<0.001
	No	29 044 (9.1)	14 327	1141 (8.0)		14 717	1503 (10.2)	
Total		33 635 (12.0)	16 850	1803 (10.7)		16 785	2231 (13.3)	

PE – physical exercise

*P-value for intra-variable difference.

## RESULTS

### Prevalence of suicidal ideation

As shown in **Table 2**, overall, 12.0% reported suicidal ideation in the past year, higher in girls (13.3%) than boys (10.7%) (χ^2^ = 53.50, *P* < 0.001). Suicidal ideation was more common among students living without both parents (16.6%), who reported high academic pressure (23.6%), who had tried smoking (19.1%), who reported binge drinking (22.7%), who had fought more than once (23.3%), who considered (27.2%) or attempted to run away from home (37.6%), who had been a victim of bullying (25.6%), and who reported often or always feeling lonely (33.1%), or sad/hopeless (30.3%).

### Univariate associations between suicidal ideation and risk factors by gender

**Table 2** summarizes the prevalence of suicidal ideation overall and comparisons between different levels of sociodemographic factors and health-risk behaviors by gender. Suicidal ideation was associated with almost all health risk behaviors and sociodemographic variables other than school type. By grade level and gender, boys in senior high school and girls in junior high school were more likely to report suicidal ideation. The students living with both parents were less likely to report suicidal ideation. Among both genders, suicidal ideation was associated with high academic pressure, lower average academic achievement, smoking, binge drinking, fighting more than once, considering or attempting running away from home, being bullied, often or always feeling lonely and feeling sad/hopeless. In general, the associations with suicidal ideation were stronger for girls than for boys.

### Multivariate associations between suicidal ideation and risk factors

According to the multivariate logistic regression, patterns of the association between suicidal ideation and risk factors were different by gender. For example, being in junior high school, ever smoking, and fighting one time were associated with suicidal ideation for girls but not boys. Junior girls were at a 24% higher risk of having suicidal ideation than senior girls (AOR = 1.24, 95% CI = 1.11-1.38). Girls who ever smoked were at a 52% higher risk of having suicidal ideation than girls who did not smoke (AOR = 1.52, 95% CI = 1.33-1.72). Girls who had fighting experience were more likely to have suicidal ideation. Among boys only, having 2 or more PE classes per week was associated with less suicidal ideation (AOR = 0.75, 95% CI = 0.61-0.92) and girls showed no association between PE classes and less suicidal ideation. However, there were still a set of behavioral risk factors associated with suicidal ideation both in boys and girls, such as family structure, academic pressure, binge drinking, fighting, considering or attempting running away from home, victim of bullying, feeling lonely and being sad/hopeless. Moreover, almost all significant variables presented reliability by PRSE (**Table 3**).

**Table 3:** Multivariate logistic regression analysis between suicidal ideation, grade, family structure and health-risk behaviors by gender among Chinese students (AOR, 95% CI)

variables	Boys*	PRSE	Girls*	PRSE	Interaction term *p-value*
Grade (junior vs senior)	–		1.24 (1.11, 1.38)†	26.0	0.002
Family structure:					
Both parents	1.00		1.00		
Others	1.31 (1.14, 1.50)‡	26.0	1.17 (1.03, 1.34)†	42.2	0.271
Academic pressure:					
Low	1.00		1.00		
Average	0.95 (0.79, 1.12)	156.1	1.19 (0.97, 1.47)	60.5	0.051
High	1.55 (1.30, 1.85)‡	20.8	2.42 (1.95, 3.00)‡	12.4	<0.001
Academic achievement:					
Above average	1.00		–		
Average	0.88 (0.75, 1.02)	59.5	–		0.253
Below average	1.04 (0.90, 1.19)	194.6	–		0.459
Not sure	1.39 (1.10, 1.75)†	36.4	–		0.071
Smoking	–		1.52 (1.33, 1.72)‡	15.9	<0.001
Binge drinking (yes)	1.17 (1.01, 1.34)†	46.8	1.30 (1.09, 1.54)†	33.6	<0.001
Fighting:					
Never	1.00		1.00		
Once	1.06 (0.90, 1.25)	150.9	1.63 (1.35, 1.97)‡	19.8	<0.001
Twice or more	1.42 (1.23, 1.62)‡	20.2	1.57 (1.28, 1.92)‡	23.4	0.046
≥2 PE classes per week	0.75 (0.61, 0.92)†	36.5	–		<0.001
Exercising over 60 min >2 days per week	–		–		–
Considering running away from home	3.70 (3.27, 4.19)‡	4.8	3.15 (2.82, 3.52)‡	4.9	0.770
Running away from home	1.87 (1.59, 2.20)‡	13.4	1.72 (1.42, 2.07)‡	17.8	0.735
Victim of bullying	1.49 (1.31, 1.71)‡	17.0	1.54 (1.31, 1.82)‡	19.1	0.226
Feeling lonely:					
Never	1.00		1.00		
Sometimes or seldom	1.49 (1.27, 1.75)‡	20.3	1.53 (1.30, 1.80)‡	19.5	0.199
Often or always	3.00 (2.50, 3.59)‡	8.5	3.40 (2.83, 4.10)‡	7.8	0.008
Being sad/hopeless	2.04 (1.79, 2.33)‡	9.4	2.39 (2.10, 2.72)‡	7.5	0.018

PRSE – percent relative standard error, AOR – adjusted odds ratio, CI – confidence interval, PE – physical exercise

*Adjusted for school type in all models. – designates not entered into the model.

†*P* < 0.05.

‡*P* < 0.001.

### Interaction effects on suicidal ideation between gender and behavioral risk factors

As shown in **Table 3**, girls showed stronger associations with suicidal ideation (with higher AORs) for junior high school (1.24 vs NA), high academic pressure (2.42 vs 1.55), and almost all health risk behaviors, such as smoking (1.52 vs NA), binge drinking (1.30 vs 1.17), fighting once (1.63 vs 1.06), feeling lonely often or always (3.40 vs 3.00), and being sad/hopeless (2.39 vs 2.04), and their interaction with gender were all statistically significant (*P* < 0.05), but considered running away from home was the exception, which was a stronger correlate for boys (3.70 vs 3.15).

## DISCUSSION

In this study, 12.0% of students in Beijing reported suicidal ideation in the past year, which is lower than the Chinese average for youth (17.99%) from 2002-2012 [26]. Our study is consistent with previous researches in Australia and American [7,27,28] that girls had a higher risk of suicidal ideation than boys. However, girls from Australian secondary schools showed much higher risk of suicide ideation than boys (21.8% vs 8.3%) [7]. In our study, girls in junior high school were more likely than boys and senior high school girls to report suicidal ideation, which may relate to the early-maturing of female students. Ge et al. found [29] that early-maturing girls represented the group with the highest rate of depressive symptoms and had more possibility of suicide ideation.

Residing with two parents was associated with a lower likelihood of suicidal ideation. Xing et al. reported [30] that for many children with divorced parents or parents working out of town for extended periods, social resources within the families are reduced. Garnefski and Diekstra [31] observed that the lowest risk of suicide attempts was in the intact family structure compared with others. They found that students from one parent and stepparent families reported more emotional problems, ie, lower self-esteem, more symptoms of anxiety and loneliness, more depressed mood and more suicidal thoughts than children from intact families, especially for girls [31]. In the present study, 18.3% of girls in non-intact families reported suicidal ideation.

Our findings showed that academic pressure and achievement showed strong association with suicidal ideation for boys and girls. In China, college entrance and senior high school entrance examinations (named “gaokao” and “zhongkao”) decide whether students can enter a key university or senior high school. Emphasizing academic success and very high academic expectations have become common concerns of Chinese parents [32,33]. Consequently, Chinese high school students spend a great deal of time on school assignments and in preparation for examinations, which may contribute to mental health disorders and suicidal behaviors [33,34]. In the current study, girls not only showed higher academic pressure but also were more likely to report suicidal ideation at the same pressure or achievement levels as boys. According to Ge et al.’s theory [29], girls were more likely than boys to encounter vulnerability when confronted with stress. This may be due to gender differences in family and social roles, social status, and problem-solving [35]. Moreover, Chinese adolescents are more suffering from the academic pressure than those in Western countries because of the different cultural background, and the evidence of migrant studies also showed that Asian American had a higher prevalence of suicide plan or attempts than other races due to the academic pressure in most cases [36].

Prior researches [18,37] have found that in young people and adults, smoking is among the strongest predictors of suicidal behavior [38] in the Western. But in the present study, smoking was only associated with suicidal ideation for girls. Moreover, binge drinking and fighting experience were more strongly associated with suicidal ideation for girls than boys in this study. In the context of Chinese traditional culture, smoking, binge drinking and fighting among girls are disapproved of by adults and society [20]. Girls having had these experiences or behaviors may be considered rebellious and may experience alienation. Prohibition of alcohol, tobacco and campus violence are imperative for school management and may provide a protective influence on suicidal behavior.

Both boys and girls with behavioral risk factors like considering or attempting running away from home and being bullied were at higher risk of suicidal ideation in our study, which is consistentwith previous studies [39,40]. Being bullied can be a significant traumatic stress, and associated with many negative effects [41-44]. The present findings indicated that about 11.7% students ever experienced bullying, higher in boys (16.3%) than girls (7%). While girls were less likely to be bullied, girls who were bullied showed a much higher prevalence of suicidal ideation than boys bullied (33.2% vs 22.4%). Bannink et al. reported that traditional as well as cyber bullying victimization is associated with an increasing risk of mental health problems among girls [43]. The gender difference in strength of association between bullying and suicidal ideation may be partly explained by differences in the types of bullying to which boys and girls are exposed. Previous studies have found that girls often experience more relational victimization (eg, spreading rumors or social isolation), and that relational victimization has a greater impact on mental health problems than overt, physical victimization, which is more often experienced by boys [45-47]. Our findings stress the importance of programs aiming at reducing bullying behaviors in schools, both for girls and boys.

Many studies show that depression is an essential mediator between suicide and health risk factors [44]. In this study, boys reported a slightly higher prevalence of depressive symptoms, eg, feeling so sad or hopeless almost every day for 2 weeks or more in a row that you stopped doing some usual activities; however, girls with depressive symptoms had the highest suicidal ideation prevalence. These girls may need more psychological assistance and supports in daily life. We found that having two or more PE classes per week was associated with a lower likelihood of suicidal ideation among boys, but not girls. Exercising over 60 minutes more than twice a week showed a weak association with boys’ suicidal ideation. Research [48-50] has consistently demonstrated that exercise is associated with lower levels of clinical and subclinical depression in cross-sectional and experimental studies. Further, exercise has been found to have a direct negative association with suicide risk and also an indirect relation with suicide risk through depression symptom and sleep quality [51]. That is, higher levels of exercise were associated with lower depressive symptoms and better sleep quality, which were in turn was associated with less suicide risk. Promoting exercise before, during (though daily PE), and after school could be a simple and realizable intervention with broad physical and mental population health benefits [52-54].

### Limitations

Overall, the findings demonstrated consistent and strong associations between health-risk behaviors and suicidal ideation for boys and girls in Beijing junior and senior high schools. Several limitations ought to be considered when interpreting the findings. First, because of the cross-sectional study design, we could only demonstrate associations and not causal paths between suicide and health risk factors. Second, the traditional view that suicidal ideation and depression represent unhealthy psychological status may have biased reporting and resulted in an underestimate of suicidal ideation prevalence. Third, due to the highly developed socioeconomic status of Beijing, not all presenting findings are suitable for extrapolating to other underdeveloped regions in China, and more researches about these regions are needed.

## CONCLUSIONS

Academic pressure and achievement, family structure, substance use, fighting or being bullied, runaway behaviors and mental health problems are associated with suicidal ideation for boys and girls. Girls were more likely to report suicidal ideation than boys. By gender and grade, girls in junior high school were the most likely to report suicidal ideation. With a great deal of emotional investment in one’s offspring, magnified by China’s single-child policy, efforts to address suicidal ideation and prevent suicide seem imperative for Chinese society and school administers. School health providers and parents ought to be mindful of the signs and correlates of suicidal ideation. Regular physical education and health education on mental health supported by campus psychologists might help to prevent suicide ideation and mental health problems in Chinese youth.
